# Plasma Enhanced High-Rate Deposition of Advanced Film Materials by Metal Reactive Evaporation in Organosilicon Vapors

**DOI:** 10.3390/membranes13040374

**Published:** 2023-03-24

**Authors:** Andrey Menshakov, Yulia Bruhanova, Polina Skorynina, Anatoliy Medvedev

**Affiliations:** 1 Institute of Electrophysics of the Ural Branch of the Russian Academy of Sciences, 106 Amundsen St., Yekaterinburg 620016, Russia; 2 Institute of Physics and Technology, Ural Federal University, 19 Mira St., Yekaterinburg 620002, Russia; 3 Institute of Engineering Science of the Ural Branch of the Russian Academy of Sciences, 34 Komsomolskaya Str., Yekaterinburg 620049, Russia

**Keywords:** TiSiCN, nanocomposite coatings, film membranes, PECVD, PVD, hollow cathode arc, anodic evaporation, ion assistance

## Abstract

Dense homogeneous nanocomposite TiSiCN coatings with a thickness of up to 15 microns and a hardness of up to 42 GPa were obtained by the method of reactive titanium evaporation in a hollow cathode arc discharge in an Ar + C_2_H_2_ + N_2_-gas mixture with the addition of hexamethyldisilazane (HMDS). An analysis of the plasma composition showed that this method allowed for a wide range of changes in the activation degree of all components of the gas mixture, providing a high (up to 20 mA/cm^2^) ion current density. It is possible to widely change the chemical composition, microstructure, deposition rate, and properties of coatings obtained by this method, by changing the pressure, composition, and activation degree of the vapor–gas mixture. An increase in the fluxes of C_2_H_2_, N_2_, HMDS, and discharge current leads to an increase in the rate of coating formation. However, the optimal coatings from the point of view of microhardness were obtained at a low discharge current of 10 A and relatively low contents of C_2_H_2_ (1 sccm) and HMDS (0.3 g/h), exceeding which leads to a decrease in the hardness of the films and the deterioration of their quality, which can be explained by the excessive ionic exposure and the non-optimal chemical composition of the coatings.

## 1. Introduction

One of the attractive promising materials are films based on silicon carbonitride, which, depending on the methods and conditions of synthesis, may have specific properties and find application in various fields. For example, in the field of microporous membrane materials, membranes based on hydrogenated silicon carbonitride a-SiC_x_N_y_:H, obtained by the PECVD (Plasma Enhanced Chemical Vapor Deposition) method, can be used to separate small gas molecules such as helium and hydrogen [1]. Such membranes are particularly attractive for H_2_ extraction, since they combine the mechanism of molecular sieving with thermally activated microporous diffusion and at the same time increase the stability of steam compared to pure silica membranes [2], which can be used in hydrogen energy technologies when creating hydrogen fuel cells. In addition, amorphous a-SiCN coatings can have good dielectric properties, chemical inertia, and high mechanical strength [3,4], and nanocomposite coatings based on the SiCN matrix can have unique combinations of such characteristics as high thermal resistance, oxidation resistance, and high hardness, etc. [5,6], due to which they can be used as protective coatings in extreme operating conditions. The properties of film materials, such as membranes or protective coatings, are determined mainly by the method and synthesis conditions. Therefore, the development and research of approaches that provide flexible modification of various synthesis conditions, allowing the creation of materials with specified properties, is an urgent task.

One of the promising SiCN-based materials are TiSiCN coatings. These films have a nanocomposite structure consisting of an amorphous SiCN matrix and embedded TiCN or TiC/TiN nanocrystals. This compound has a unique combination of physicochemical and mechanical properties, such as high hardness [7], heat resistance [8], oxidation resistance [9], a low coefficient of friction [10], and biocompatibility [11]. It makes TiSiCN a promising protective wear-resistant coating for various applications from biomedicine to aerospace applications [12,13,14]. There are a number of methods for obtaining such coatings [15], and, in our opinion, the combined plasma chemical methods using organosilicon compounds (OSCs) as a source of silicon in the coating are advanced and promising methods [16,17,18]. This work is devoted to the study of optimal conditions for the synthesis of such coatings. To obtain nanocrystalline and nanocomposite coatings by vacuum-plasma methods, it is necessary to create conditions that ensure a sufficiently high migration mobility of atoms on the surface of the growing coating [17]. With a decrease in the temperature of the substrates or an increase in the coating rate, such conditions are achieved by increasing the intensity of ion assistance [18]. As a rule, strict restrictions are imposed on the range of ion energies bombarding the coating during its growth, due to a decrease in the proportion of the coating material in the required structural-phase state [19,20,21] and the development of internal stresses that worsen adhesion [22]. In this connection, low-energy ion assistance seems promising, in which the required average value of ion energy per coating atom, defined as the product of E_i_ * j_i_/j_0_ (where E_i_, j_i_ are the energy and current density of ions, j_o_ is the density of the flow of atoms), is achieved by increasing j_i_. The required value of j_i_ (~10 mA/cm^2^ or more) under conditions of high-rate deposition (~10 µm/h) is provided by ionization of the flow of particles forming the coating [23], or by an increase in the current density of gas ions coming from the auxiliary discharge plasma [24,25].

Dense and hard crystalline coatings are formed at a sufficiently high ratio of the ion flux to the neutrals j_i_/j_0_ on the substrate [18]. Therefore, when coatings are obtained by magnetron sputtering or evaporation by an electron beam with a high deposition rate, a high j_i_/j_0_ ratio is often achieved by additional gas ionization [26]. In a vacuum-arc discharge, the j_i_/j_0_ ratio usually is up to 10% and a high deposition rate of up to 50 µm/h is provided at currents from 100 A and above [27]. For many applications, the disadvantage of the method is the presence of a micro-droplet fraction in the plasma stream, the elimination of which by additional filtration systems leads to a several-fold decrease in the deposition rate, energy, and ion concentration. Another disadvantage of this method is a sufficiently wide energy spectrum of metal ions, whereas precise energy control is necessary for the synthesis of the desired crystalline phase.

The disadvantages above describe the deprivation of obtaining coatings by reactive anodic evaporation of titanium and decomposition of an OSC precursor in a discharge with a self-heating hollow cathode (SHHC). The possibility of obtaining TiSiCN coatings by the proposed method was realized in [28], where the results of the first experiments are presented, showing the fundamental possibility of obtaining nanocomposite TiSiCN coatings by this method. In such a discharge, a high-density plasma is created without using a separate source of vapor–gas mixture ionization, and filtration systems are not used, since there are no microdrops in the vaporized metal stream. The electron flow, in which there is a high proportion of fast electrons with an energy of up to several tens of eV [29], has a higher ability to dissociate and ionize the components of the gas mixture. The advantage of this approach is also that the formation of a vapor stream of titanium and dense plasma, as well as the decomposition of precursor molecules, occurs in one discharge, while the composition of the vapor–gas mixture, the density of the ion current, and the evaporation rate of titanium can vary independently and over a wide range using a sectional anode.

One of the advantages of using OSCs is that the synthesis of films involves not individual atoms, but fragments of molecules formed as a result of the decay of the original molecules in plasma and already containing Si–C and Si–N bonds, which facilitates the formation of the SiCN matrix. Therefore, it is important to control the decomposition degree of the precursor by limiting the discharge power during the synthesis of coatings. Thus, it is necessary to ensure a balance of deposition conditions under which, on the one hand, a high ion current density is provided, ensuring the formation of the desired crystalline phase at a sufficiently high rate, and on the other hand, the discharge power should not be too high to preserve fragments of OSC molecules to facilitate the formation of TiSiCN with superior characteristics.

In this paper, using the example of TiSiCN, the potential of the considered deposition method for controlling the properties of the obtained films is demonstrated. This work is devoted to the study of the conditions for obtaining nanocomposite coatings by reactive Ti evaporation in Ar/N_2_/C_2_H_2_ plasma of a high-current discharge with the addition of hexamethyldisilazane vapors under conditions of intensive ion assisting. The paper presents the results of a study on the influence of plasma generation conditions, in particular, the pressure and composition of the gas mixture, as well as the discharge current on the ion current density, the ion composition of the plasma, and the activation degree of the components of the gas mixture during the evaporation of titanium in the reaction medium; it also examines the influence of these factors on the deposition rate and properties of TiSiCN coatings obtained by the described method.

## 2. Materials and Methods

**Figure 1:**
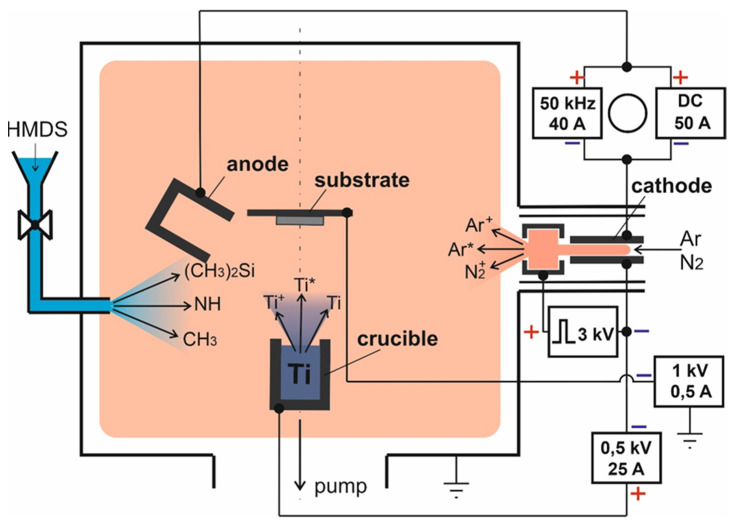
The electrode scheme of the experimental facility.

The experiments were carried out in a discharge system based on a discharge with an SHHC, the scheme of which is shown in Figure 1, and the design of the system is described in detail in [28].

A feature of the discharge system is that the discharge from the SHHC was closed to a two-section anode. One section made of stainless steel had water cooling, and the second section of the anode was a crucible (graphite MPG-7) with a Ti-hitch (VT1-0). This scheme makes it possible to independently change the currents in the crucible and anode circuits, adjusting both the current density and the amount of metal vapor flow. For better thermal insulation and to ensure high power density on the evaporated surface, the crucible is placed in heat shields made of molybdenum and ceramic tube. The discharge current was closed to the open end surface of the crucible with a diameter of ~16 mm.

The discharge was ignited in an argon medium (Q_Ar_ = 40 cm^3^/min) between the cathode and the cooled anode. Initially, the hollow cathode was heated in a pulsed-periodic glow discharge and its transition to a thermionic emission mode characterized by low voltage and a diffuse form of discharge. The discharge current to the cooled anode I_d_ was regulated in the range of 10–50 A, while the discharge voltage was 80–110 V. Subsequently, part of the I_cr_ discharge current was switched to the crucible by applying a voltage. The temperature of the crucible and the evaporation rate of titanium were regulated by changing the I_cr_ in the range of 0–10 A.

The saturation ion current density was measured using a flat one-sided probe with an area of 1 cm^2^ located near the sample holder. The temperature of plasma electrons in the same region was determined by probe diagnostics using a Langmuir probe with a length of 5 mm and a diameter of 0.4 mm, made of tungsten wire and shielded from the flow of fast electrons.

A liquid organosilicon precursor, hexamethyldisilazane (HMDS, [(CH_3_)3Si]_2_NH), was used as a silicon source for the synthesis of coatings because it is one of the most accessible and safe OSCs, which is important in the mass synthesis of coatings for industrial applications. Despite the fact that all the elements necessary for the synthesis of TiSiCN are present in the vapor–gas mixture of the titanium and hexamethyldisilazane vapors, the properties of such coatings, in particular their hardness, wear resistance, and the coefficient of friction, are affected by the content of carbon and nitrogen. For example, if the carbon content in TiSiCN is higher than the amount that can react with other elements, excess carbon is deposited in the form of amorphous or diamond-like carbon (DLC) nanoparticles [30]. The presence of self-lubricating components in the microstructure of superhard TiSiCN coatings can significantly reduce its coefficient of friction. Due to the combination of a low coefficient of friction and high hardness, TiSiCN coatings can provide excellent wear resistance compared to conventional nitride or carbonitride coatings. The nitrogen content also affects the relative content of nitride and carbonitride phases, and, accordingly, the hardness and wear resistance of the coatings. Therefore, for a more accurate selection of the content of each element in the coating, reactive gases containing N and C are introduced into the gas mixture. In the discussed experiments, nitrogen and acetylene gas were used for these purposes, the latter having the highest relative content of carbon atoms in the molecule.

The flow of HMDS vapors was fed into the chamber through an evaporator located at a distance of 10 cm from the sample holder. The vapor flow was regulated by a Mini Cori-Flow digital mass flow controller (Bronkhorst, Ruurlo, The Netherlands) in the range of 0–10 g/h. The C_2_H_2_ and N_2_ were injected through a cooled hollow anode, a supply method which ensured that the reactive gases achieved intensive activation due to the combination of increased pressure in the anode cavity and increased plasma concentration near the anode.

Before the start of the experiment, the AISI304 austenitic stainless steel samples were cleaned in an ultrasonic bath in acetone for 20 min, then dried and placed on an insulated holder located above the crucible at a distance of 8 cm. The residual pressure in the vacuum chamber at the beginning of the treatment cycle was about 2 × 10^−2^ mTorr. Before the deposition cycle, ion purification of the samples was carried out for 10 min using Ar ions with an energy of 500 eV. After purification, the energy decreased to 80–100 eV. To improve the adhesion of the coating to the substrate, a Ti sublayer with a thickness of ~100–200 nm was first applied, after which nitrogen (10 cm^3^/min) was supplied, and to ensure a smoother gradient of properties from the substrate to the hard coating, an intermediate TiN sublayer of the same thickness was deposited, following which hexamethyldisilazane and acetylene were injected into the chamber. This sublayer structure ensured good adhesion of hard TiSiCN coatings to the substrate in all the studied synthesis modes. 

The plasma composition was analyzed by optical emission spectroscopy using an OceanOptics HR2000 spectrometer in the wavelength range from 200 to 1100 nm, with a resolution of 0.84 nm. The thickness of the coatings was determined by abrasion with a steel (VK6) ball of the sample surface on a Calotest device (CSM Instruments) with an accuracy of 0.1 microns. The hardness of the obtained films was measured by microindentation on a dynamic SHIMADZU DUH-211S microhardness meter with a load of 20 mN. The average of 15–20 measurements was used. The chemical composition of the coatings was studied using a Tescan VEGA II XMU scanning electron microscope at a pressure of 10^−5^ Torr with an INCA ENERGY 450 X-ray energy dispersive microanalysis system. The phase composition was studied by X-ray diffraction (XRD) on a D8 DISCOVER diffractometer (Bruker AXS) in copper Ka1 radiation using a graphite monochromator on a secondary beam. Diffractograms were processed using the TOPAS-3 program.

## 3. Results and Discussion

**Figure 2:**
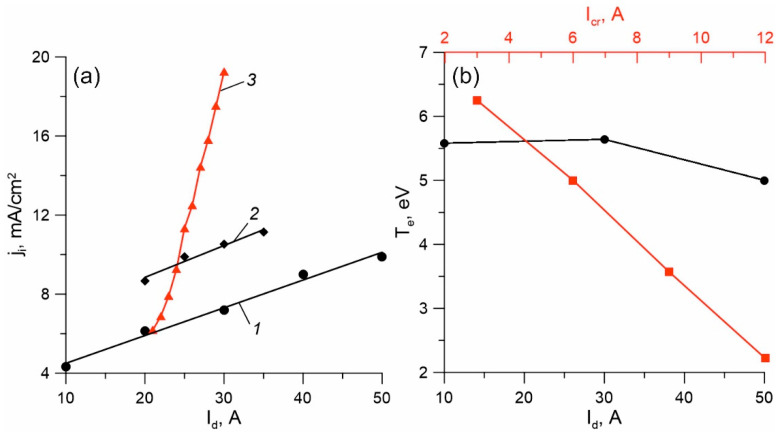
(**a**) The dependence of the saturation ion current density on the total discharge current in the argon medium: 1—without switching on the crucible; 2—I_cr_ = 5 A, current to the anode 15–30 A; 3—current to the anode 20 A (red), current in the crucible circuit 0–10 A. (**b**) The dependence of the electronic temperature on the currents in the crucible (red) and cooled anode (black).

Measurements of the saturation ion current density j_i_ showed that when the crucible was switched on, the current density on the processed samples significantly increased (Figure 2a), and an increase in the current of the crucible circuit resulted in a significant increase in the rate of growth of the ion current density (curve 3). In the absence of a current in the crucible circuit, an increase in the discharge current to I_d_ = 50 A led to an increase in the ion current density of up to 10 mA/cm^2^ (curve 1). At an I_cr_ = 5 A current in the crucible circuit, the same value of the ion current density was achieved at half the total current I_d_ = 25 A (curve 2). The density of the ion current in this case reached ~20 mA/cm^2^. The measurement of the evaporation rate of titanium showed that by changing the power supplied to the crucible surface from ~400 to ~900 W (I_cr_ = 10 A), the titanium vapor flow was regulated in the range of ~10^−5^–10^−4^ g/s cm^2^, which corresponded to the local pressure of saturated vapors near the crucible surface from ~0.3 to ~5 Pa. An increase in the current of the crucible circle in the range of 2–10 A led to a significant decrease in the temperature of the electrons, T_e_, from 6.3 to 2.2 eV (Figure 2b).

This behavior of T_e_ can be explained both by an increase in the metal component of the plasma, which is characterized by a lower value of the electron temperature [31], and by a significant increase in the pressure of the vapor–gas mixture caused by an exponential increase in the vapor pressure of the metal with an increase in the heating power and temperature of the crucible. When the I_d_ current changes in the range of 10–50 A, such a significant change in the temperature of the electrons and the plasma potential does not occur, which can be explained by the absence of a significant change in the proportion of metal vapors and their pressure.

**Figure 3:**
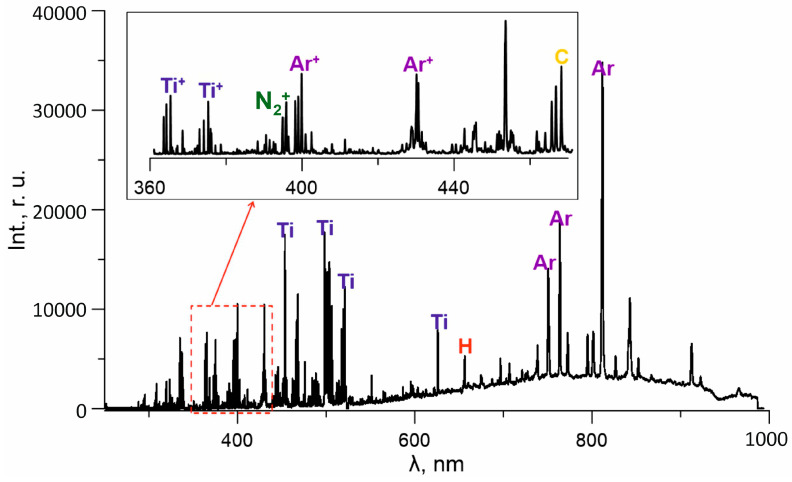
The optical spectra of the discharge plasma.

Figure 3 shows the characteristic spectrum of plasma in a mixture of argon, nitrogen, titanium, acetylene, and HMDS vapors. In all discharge modes, lines of plasma-forming gases (Ar* and Ar^+^), lines of excited titanium atoms (Ti*-399.9, 453.3, 498.2, and 504 nm) and ions (Ti^+^-337.3, 364.3, and 374.2 nm), and molecular nitrogen in excited (N_2_* 357.3 nm) and ionized (N_2_^+^ 391.4 nm) forms are observed in the spectra. In addition, in the visible part of the spectrum, there are lines of the hydrogen atom characteristic of the Balmer series (H* 656.3 nm), as well as bands of atomic carbon (C* 467.6 nm), which indicate the intensive decomposition of HMDS and C_2_H_2_ molecules in gas-discharge plasma. Thus, the ionic composition of plasma is represented by Ar^+^, Ti^+^, and N_2_^+^ ions. In contrast to the research in [32], which demonstrated that the average charge of titanium ions coming from a low-pressure arc with a cold cathode was 2.1, the lines of double-charged Ti_2_^+^ ions in the discharge plasma with the SHHC against the background of intense Ti* and Ti^+^ lines could not be distinguished. Perhaps this is due to other ionization conditions in the discussed discharge system, when titanium vapors are ionized by a stream of fast electrons with an energy of several tens of eV [29] coming from the SHHC and additionally accelerated in a double layer of spatial charge near the crucible surface, as in [33]. If one judges the proportion of titanium ions on the surface of the samples by the increment of the ion current during metal evaporation (Figure 2a, curve 3), then at a fixed current to the cooled anode I_d_, an increase in the current I_cr_ to the crucible from 0 to 10 A would increase the proportion of the current of metal ions to the samples to ~90%. However, the analysis of the plasma composition showed that the growth of the ion current with an increase in the current per crucible was not only due to an increase in the number of titanium ions, but also to an increase in the ionization degree of the gas components of the plasma.

**Figure 4:**
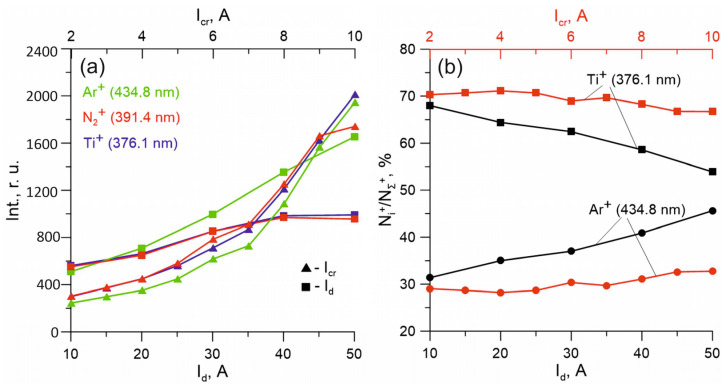
(**a**) The relative dependence of the ion line intensities and the (**b**) N^+^_i_/N^+^_Σ_ content of titanium and argon ions in plasma, on the current to the crucible (red) and to the cooled anode (black).

Figure 4a shows the relative dependence of the intensities of the argon Ar^+^, titanium Ti^+^, and nitrogen N_2_^+^ ion lines on the current to the cooled anode I_d_ and on the current to the crucible I_cr_. The results show that an increase in the discharge current from 10 to 50 A leads to a significant increase in Ar^+^ lines, but to a lesser extent the intensity of Ti lines depends on I_d_. This indicates that the activation processes of metal vapors occur mainly in the crucible region and depend to a much greater extent on the value of I_cr_ rather than I_d_. 

If the increase in the ionic current with an increase in the I_d_ current is mainly due to an increase in the number of argon ions Ar^+^, then the increase in the ionic current with an increase in the current to the crucible is not solely due to the influx of metal ions. An increase in the current to the crucible and the concentration of titanium vapors also resulted in a significant increase in the number of gas ions (Ar^+^, N_2_^+^), which can be explained by the intensification of the processes of recharging titanium ions on atoms and molecules of plasma-forming gases with an increase in the local vapor pressure of the metal in the sample location. This process can explain the acceleration of the growth of the ion current density with an increase in the current to the crucible.

The process of the appearance and departure of particles in plasma is described by the kinetic equation: $\frac{dn_{i}}{dt}=N_{i}\sigma_{0i}v_{e}-\frac{n_{i}}{\tau_{i}}$, where N_i_ is the concentration of particles in the ground state, σ_0i_ is the excitation cross section of the energy state, v_e_ is the electron velocity, τ_i_ is the lifetime of the state, and n_i_ is the concentration of particles in the excited state. In this case, n_i_ is related to the intensity of radiation in the spectrum by the Einstein ratio: I = a·hv_ki_·n_i_·A_ki_, where I is the intensity of the spectral line, a is the correction factor, hv_ki_ is the transition energy, and A_ki_ is the Einstein coefficient. In all experiments, the relative content of molecular nitrogen ions N_N2_^+^/(N_Ti+_ + N_Ar+_ + N_N2+_) in the ion stream, estimated by the ratio of spectral line intensities using the Einstein ratio, did not exceed 1% at nitrogen flows of ~10 cm^3^/min, at which most coating deposition experiments were carried out. At an argon pressure in a vacuum chamber of 0.6 mTorr, the proportion of Ti^+^ ions with an increase in the current I_cr_ to the crucible from 2 to 10 A with a constant current to the cooled anode section I_d_ = 10 A, did not change and was ~70% (Figure 4b); however, the increase in the current I_d_ from 10 to 50 A, and at constant current I_cr_ = 5 A, led to a decrease in the proportion of metal ions to ~55%. This behavior of the ionic components can be explained by the fact that the ionization of titanium vapor occurs mainly near the crucible in the region of the maximum vapor pressure of the metal and the maximum concentration and energy of the oncoming electron flow, while the vapor ionization degree is very high. Therefore, an increase in I_d_ does not lead to an increase in the ionization degree of titanium, and with an increase in the ionization degree of argon, the proportion of metal ions at a distance from the crucible decreases. An increase in argon pressure to 2 mTorr at a constant crucible current also leads to a decrease in the proportion of metal ions to ~25–30%, which can be explained not only by an increase in the concentration of Ar, but also by the scattering of the titanium vapor flow in the gap between the crucible and the substrate.

**Figure 5:**
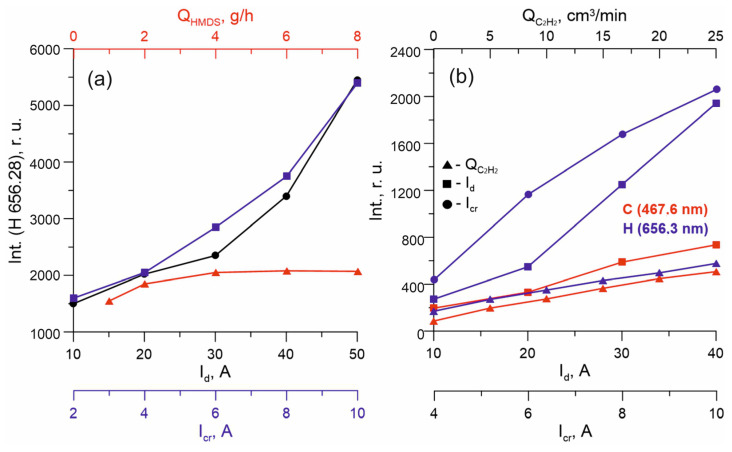
(**a**) The dependence of the intensities of line H on the discharge current I_d_ (black), the crucible current I_cr_, and the HMDS flow Q_HMDS_ (red). (**b**) The dependence of the intensities of lines C and H in the medium C_2_H_2_ on the discharge current I_d_, the crucible current I_cr_, and the flow C_2_H_2_ Q_C2H2_.

The decomposition of precursor molecules in a gas-discharge plasma is determined, first, by the binding energies between the atoms, and second, by the discharge power. The decomposition of hexamethyldisilazane in plasma is a stepwise reaction [34]: first, the Si–C bond is broken (binding energy 3.3 eV) and the methyl group is lost ((CH_3_)_3_Si]_2_NH→(CH_3_)_3_-Si-NH-Si-(CH_3_)_2_ + CH_3_). Subsequently, the Si–N (binding energy 3.5–3.7 eV) single bond is broken and fragments of a molecule with a double bond Si=N appear in the vapor–gas medium: (CH_3_)_3_-Si-NH-Si-(CH_3_)_2_ → (CH_3_)_3_-Si + HN = Si-(CH_3_)_2_. A further increase in the discharge power leads to a deeper precursor decomposition, the bonds N–H (4–4.1 eV), C–H (4.3–4.8 eV), and Si = N are destroyed, as a result of which H, C, and Si atoms appear in the plasma. Among the decomposition products of the HMDS molecule against the background of strong argon and titanium lines, only hydrogen lines were found in spectra, the intensity of which was used to determine the activation degree of the precursor. The results of the analysis of the plasma composition indicate an increase in the decomposition degree of HMDS molecules with an increase in the discharge current (Figure 5a). 

With an increase in the current of the circuit for each anode section, the intensity of the hydrogen line, which is proportional to the content of this component in the plasma, increases almost linearly. Moreover, the enhancement of the precursor decomposition with an increase of I_cr_ is more effective, since the intensities of the H* line at the crucible current I_cr_ = 10 A are achieved only at the current I_d_ = 50 A. The more effective decomposition of the precursor with an increase in the I_cr_ current can be explained in our case by the fact that OSC vapors are released into the titanium vapor drift region, which creates conditions combining high plasma concentration and high OSC vapor density. At the same time, an increase in the vapor flow from 1 to 8 g/h at a constant discharge current does not, in practice, lead to an increase in the intensity of the H line, which indicates a decrease in the activation degree and efficiency of the use of the precursor with an increase in Q_HMDS_ at a constant value of the discharge current I_d_, and consequently, an excess of the precursor for this value of the discharge power.

Considering that mainly atomic particles participate in the formation of the carbide, nitride, or carbonitride nanocrystalline phase in the coating, a change in the dissociation degree of N_2_ and C_2_H_2_ can affect the kinetics of the deposition process.

The study of acetylene dissociation in the absence of precursor vapors showed that an increase in the discharge current led to a monotonous increase in the intensities of the decomposition product lines of the initial molecules, which probably indicates an excess of the reactive component and its incomplete decomposition. In [35] acetylene–argon, the plasma of a low-pressure arc discharge was investigated, and according to the results obtained, argon ions Ar^+^ play a key role in the dissociation of C_2_H_2_ molecules: Ar^+^ + C_2_H_2_ → Ar + C_2_H_2_^+^, C_2_H_2_^+,*^ + e → C_2_H + H (26%), → C_2_ + H + H (41%), → 2CH (7%), → CH_2_ + C (26%), → C_2_ + H_2_ (0%). The plasma spectroscopy results obtained in this work are in satisfactory agreement with [34]. A large number of argon Ar^+^ ions are also present in the discharge plasma with an SHHC and an active anode, contributing to the dissociation of acetylene. The absence of lines of other C_2_H_2_ decomposition products (in addition to H* and C*) is apparently due to the relatively low intensity of the corresponding lines against the background of strong argon and titanium lines.

The most probable mechanism for the formation of atomic nitrogen, which plays an important role in the synthesis of nitride coatings, in the presence of fast electrons in the plasma, is the direct ionization of nitrogen molecules by the electron impact with the formation of a molecular ion N_2_^+^ and subsequent dissociative recombination of molecular nitrogen ions with the participation of low-energy plasma electrons [36]. In the spectra of a vapor–gas mixture with titanium and HMDS, lines of atomic nitrogen N* against the background of intense lines of other elements are not detected, but the number of nitrogen atoms is indirectly indicated by the number of molecular nitrogen ions, according to the basic mechanism of the generation of nitrogen atoms described above. It can be seen from the results obtained that an increase in the current to both anode sections leads to a monotonous increase in the number of molecular nitrogen ions (Figure 4a). However, it should be noted that if an increase in the I_d_ current in the range of 10–50 A leads to an increase in the intensities of N_2_^+^ lines by 35–40%, then an increase in the current in the crucible circle in the range of 2–10 A and the corresponding concentration of titanium vapors leads to a more significant increase in the number of N_2_^+^ ions, which can be explained by the processes of recharging the titanium ions on the atoms and the plasma-forming gas molecules at an elevated local metal vapor pressure. In nitrogen–argon plasma, when atomic nitrogen lines were reliably detected in the spectra, the nitrogen dissociation degree was 4–6%. Thus, in a discharge system with a sectional anode, effective dissociation of all reactive components of the vapor–gas mixture is ensured, and the activation degree and the relative content of each component can be changed within a wide range by adjusting the rate of their supply and changing the discharge power.

At different values of pressure and composition of the vapor–gas mixture, TiSiCN coatings were synthesized. The N_2_ flow in the experiments was 10 sccm, and the acetylene flow was 1–4 sccm, and the relatively low content of reactive components in the gas mixture was compensated by its sufficiently high activation degree under the conditions studied.

**Figure 6:**
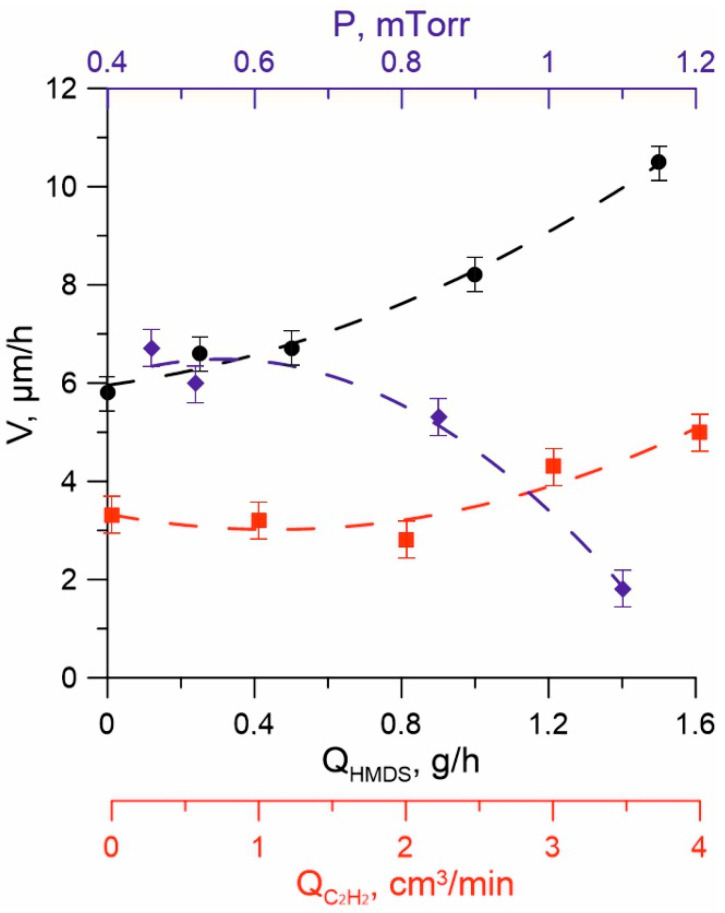
The dependence of the deposition rate of coatings on the argon pressure P (blue) at Q_HMDS_ = 1 g/h, I_d_ = 10 A, on the Q_HMDS_ flow (black) at I_d_ = 12 A, P_Ar_ = 0.45 mTorr, and the Q_C2H2_ flow (red) at Q_HMDS_ = 0.5 g/h and P_Ar_ = 1 mTorr.

The relative dependence of the deposition rate of the coatings on the gas mixture pressure and the flows of HMDS and C_2_H_2_ are shown in Figure 6. An increase in the vapor flows of the precursor and acetylene leads to an increase in the deposition rate, and if the acetylene flow in the range of 0–4 cm^3^/min has little effect, then an increase in the flow of HMDS to 1.5 g/h leads to an increase in the deposition rate from 6 to 10 µm/h at a discharge current of 20 A and a current in the crucible circle of 7 A. Pressure also significantly affects the rate of coating formation. An increase in the argon pressure in the gas mixture from 0.5 to 2.2 mTorr leads to a decrease in the deposition rate from ~6.5 to ~1.5 µm/h, which is due to an increase in the scattering of metal vapor and precursor flows in a high-pressure medium.

With an increase in the discharge current, the rate of coating formation also increases, which can be explained by an increase in the decomposition degree of the precursor and the dissociation degree of nitrogen and acetylene with an increase in plasma concentration and an increase in the intensity of plasma chemical processes in the plasma generation region. At the same time, the density of the ion current and the degree of ion impact on the surface of the growing coating increases. An increase in the I_cr_ in the range of 3–8 A in Ar medium led to an increase in the deposition rate of coatings from 1.5 to 4 microns. At the same time, an excessive increase in the current to the crucible leads to a deterioration in the quality of coatings, at a current of I_cr_ = 8 A, chipping appears on the surface of the coatings, and with a further increase in I_cr_ cracks appear and the number of chips increases, which is apparently due to an excessive increase in the level of internal stresses in the coating as a result of increased ion exposure on its surface. An increase in the energy of ions bombarding the treated surface by increasing the bias voltage over 200 V has the same effect on the quality of coatings. Reducing the ion exposure degree by reducing the bias voltage in the samples to 0 at the same I_cr_ current leads to an improvement in the structure and quality of the films. However, the hardness of such coatings is significantly reduced (to 20–22 GPa).

**Figure 7:**
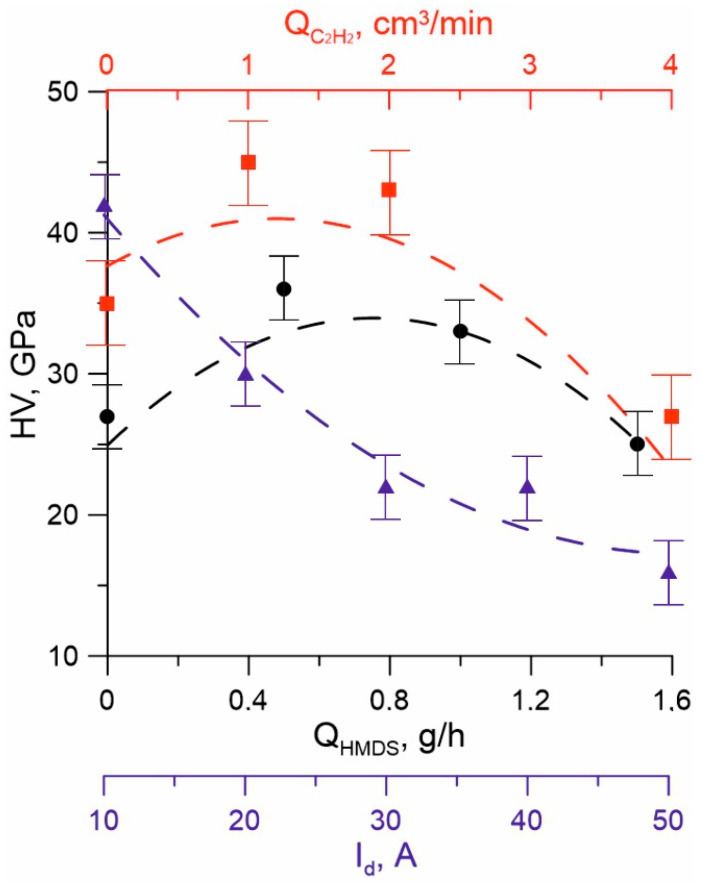
The hardness of TiSiCN coatings on the discharge current I_d_ (blue), HMDS flow Q_HMDS_ (black), and C_2_H_2_ flow QC_2_H_2_ (red).

The microhardness of coatings depends nonmonotonically on Q_HMDS_ and Q_C2H2_ (Figure 7). Thus, at the constant current values I_d_ = 10 A, I_cr_ = 6 A, Q_N2_ = 10 sccm, and Q_C2H2_ = 0 sccm, the maximum coating hardness values of 36–38 GPa were obtained at a flow of 0.25–0.5 g/h HMDS, and a further increase in Q_HMDS_ to 1.5 g/h led to a decrease in the microhardness of coatings to 25–26 GPa. The addition of acetylene to the gas mixture of 1 sccm at Q_HMDS_ = 0.5 g/h led to an increase in microhardness to 42–43 GPa. However, with a further increase in Q_C2H2_, a decrease in the hardness of the coatings was observed, as well as a deterioration in their quality. A possible reason for the decrease in microhardness with an excessive content of precursor and acetylene may be an increase in the content of the amorphous phase. An increase in the nitrogen flow from 10 to 30 sccm leads to a decrease in microhardness up to 28 GPa. Another reason for the decrease in hardness, both with an increase in the precursor flow and with an increase in the flow of nitrogen and acetylene, may be an increase in the hydrogen content in the coating. Research has shown [37] that when OSCs with a high content of atomic hydrogen were used in plasma processes, the presence of additional nitrogen in the gas phase led to the formation of Si–NH–Si fragments, which significantly reduced the hardness of the films. The hardness of the obtained coatings decreased from 30 to 5 GPa with an increase in the N_2_ content in the initial gas mixture. The authors of [38] also suggest that a possible reason for the decrease in the hardness of coatings with an increase in the proportion of nitrogen is that the concentration of Si–H and N–H bonds increases when nitrogen atoms are introduced into the film. In addition, it was demonstrated in [39] that hydrogen bound to carbon in the form of “organic” fragments of the initial compound also reduced the mechanical characteristics of films.

An increase in the discharge current I_d_ in the range of 10–50 A (Figure 7) leads to a decrease in the microhardness of the obtained films. This is probably due to a deeper decomposition of the initial precursor molecules and a decrease in the proportion of the nanocrystalline phase in the coating composition, as evidenced by the results of the X-ray phase analysis, in which a relative decrease in the intensity of the lines of nanocrystalline phases on the radiographs was observed.

The results of the chemical analysis of the composition of coatings synthesized under various conditions, presented in Table 1, are consistent with the results of measuring their microhardness. The data show that the pressure and composition of the vapor– gas mixture have a significant effect on the chemical composition of the coatings. Thus, an increase in argon pressure leads to a significant decrease in the titanium content even at a high current to the crucible, which is consistent with spectroscopy data on a decrease in the proportion of metal ions with an increase in pressure. At the same time, an increase in the current to the crucible increases the activation degree of the precursor and acetylene, which leads to an increase in the content of Si and C in the coating.

**Figure 8:**
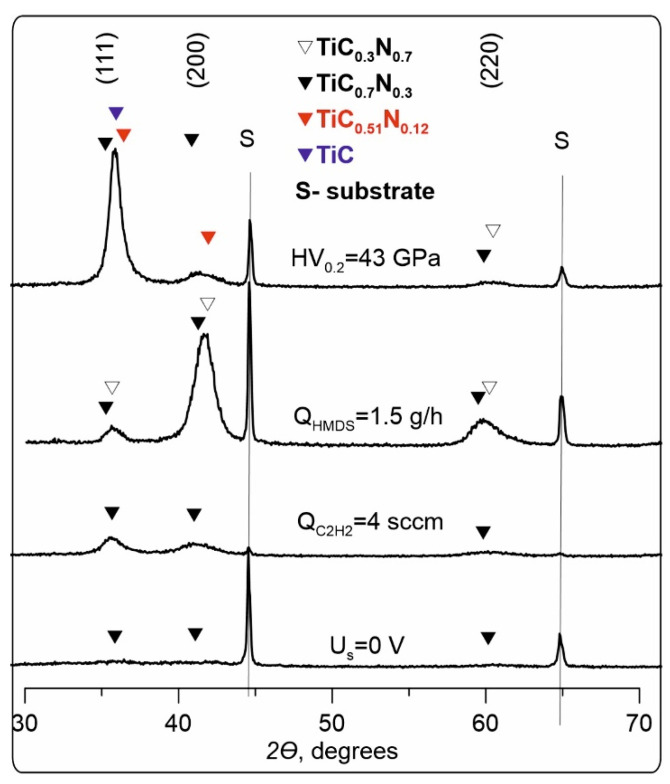
XRD patterns of TiSiCN coatings.

The results of the XRD are presented in Figure 8. The thickness of the coatings was 4–5 microns. Changing the synthesis modes and composition of coatings leads to a corresponding change in the nanocrystalline phase of coatings. In “suboptimal” synthesis modes, i.e., under conditions of excess levels of the precursor at Q_HMDS_ = 1.5 g/h, or excess acetylene (Q_C2H2_ = 4 sccm), the nanocrystalline phase of the coating consists of cubic TiC_0.7_N_0.3_ crystallites (OCD ~2–5 nm) or mixtures with the phase TiC_0.3_N_0.7_ (OCD ~6–9 nm). The coatings having the maximum obtained microhardness contain the following crystalline phases: solid solution based on TiC_0.7_N_0.3_ (PDF No. 42-1489, cubic, OCD ~20 nm), solid solution based on TiC_0.51_N_0.12_ (PDF No. 85-1152, hexagonal, OCD ~5 nm), and TiC—Khamrabaevite (or a solid solution based on it) (PDF No. 32-1383, cubic, OCD ~11 nm, preferred orientation (111)). Moreover, the intensity of the peaks on the X-ray grams is significantly higher with the same thickness of coatings, which may indicate an increased content of the nanocrystalline phase and explain the observed increased microhardness of films in comparison to the “non-optimal” synthesis conditions (Figure 7). When the displacement voltage, which determines the energy of bombarding ions, is reduced from 100 V to U_s_ = 0 V, the nanocrystalline phase consists of TiC_0.7_N_0.3_ crystals (OCD ~3 nm); however, its content in the coatings is extremely low, as evidenced by very low peak intensities on X-ray images. This may also explain the significant decrease in the microhardness of the coatings in comparison with the increased energy level of ions acting on the surface. Silicon in crystalline phases was not detected in any of the synthesis modes. This suggests that Si in the obtained coatings is present only in the amorphous state in the form of phases of carbonitride a-SiC_x_N_y_, or nitride a-Si_3_N_4_ and carbide a-Si_3_C_4_ [40,41]. This is consistent with the known data [14] and may be explained by the fact that Si_3_N_4_ crystallizes at temperatures above 1473 K [42], and β-SiC crystals form at temperatures above 1573 K [43].

**Figure 9:**
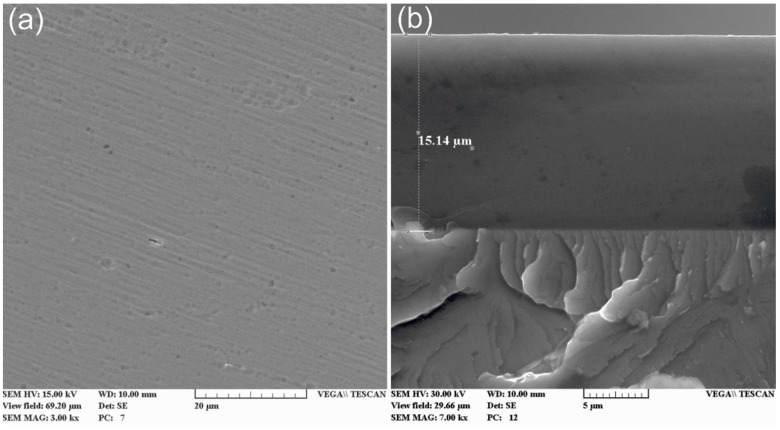
SEM images of the surface (**a**) and cross section (**b**) of a TiSiCN coating.

Figure 9 shows a characteristic image of the cross section of the coating with a thickness of more than 15 microns and a dense homogeneous structure with good adhesion to the substrate.

**Figure 10:**
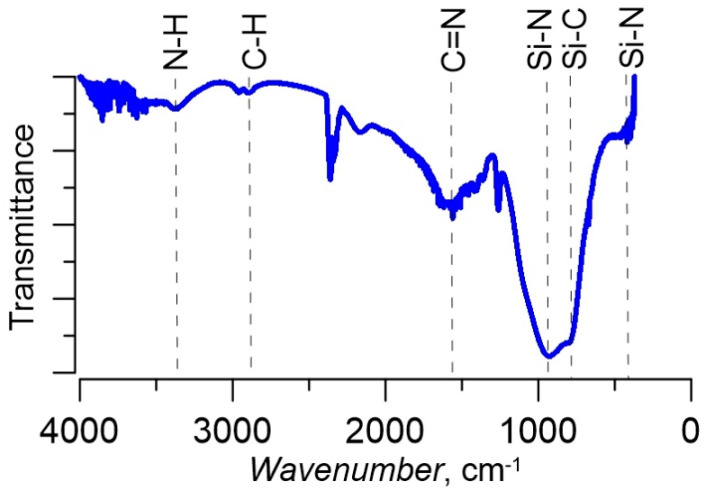
An IR-spectra of the SiCN coating.

In the discharge mode with one anode section, in the absence of a current in the crucible circle and, accordingly, in the absence of a metal vapor flow in the plasma, in the medium Ar/N_2_ at a low current I_d_ = 10 A, the test SiCN coatings were obtained. The coatings had good dielectric properties, the microhardness of the obtained films was 10–12 GPa, which is typical for low-temperature deposition of these types of coatings. XRD analysis showed that the coatings obtained in this mode are X-ray amorphous, and the results of IR spectroscopy (Figure 10) showed the presence of fragments of the original HMDS molecule, even with weak bonds, which correspond to the «soft» mode of precursor decomposition at a low discharge current.

## 4. Conclusions

The method of reactive evaporation of titanium in a hollow cathode arc discharge in an atmosphere containing organosilicon vapors makes it possible to provide a high (up to 20 mA/cm^2^) ion current density and also to change the composition of the ion flux within a wide range, to reach the proportion of metal ions up to 70%. The use of a sectional anode and the independent supply of each component forming the TiSiCN structure makes it possible to independently change the pressure and composition of the reactive gas medium, as well as its activation degree, which opens up opportunities to change, over a wide range, the composition and properties of the coatings. Hard (up to 42 GPa) nanocomposite TiSiCN coatings with a dense homogeneous structure with a thickness of up to 15 microns were obtained under conditions of intensive ion assistance at a deposition rate of 5 µm/h. The present study shows that, on the one hand, an increase in the flows of reactive components, as well as the discharge current, leads to an increase in the rate of coating formation up to 10 µm/h in the studied configuration of the gas discharge system. On the other hand, excessive growth of these parameters leads to the deterioration of both the properties and the quality of the resulting films, which is due to a number of factors from suboptimal chemical composition to excessively intense ionic effects on the surface of the growing coating. Thus, the investigated method of obtaining coatings allows not only the generation of coatings with given compositions, structures, and properties, it also allows, by changing various synthesis conditions during deposition in one working cycle, the production of layers with different chemical compositions, structures, and electrical and mechanical properties. These range from relatively simple nanocrystalline Ti/TiN coatings to more complex amorphous or nanocomposite TiSiCN coatings, so this method can be applied to other types of films, including the promising SiCN-based membranes.

## Figures and Tables

**Table 1:** The chemical composition of the surface of the samples (at. %) and the modes of obtaining coatings.

#	Q_N2_ sccm	Q_C2H2_, sccm	Q_HMDS_, g/h	P_Ar_, mTorr	I_cr_, A	nTi, %	nSi, %	nN, %	nC, %	HV, GPa	H/E
1	10	1	0.5	0.5	4	52	13.2	22.7	12.1	42	0.16
2	30	1	0.5	1	4	38	19.8	37.8	4.3	28	0,13
3	10	1	0.5	1	8	35.1	27.6	24.1	13.2	21	0.09
4	10	4	0.5	0.5	4	48.9	20.9	15.5	14.7	29	0.12
5	10	0	0.25	0.5	6	63.4	6.3	22	8.3	38	0.13
6	10	0	1.5	0.5	6	57	16.6	10.4	16	25	0.13

## Data Availability

The data presented in this study are available on request from the corresponding author.
